# Facial and Vocal Markers of Schizophrenia Measured Using Remote Smartphone Assessments: Observational Study

**DOI:** 10.2196/26276

**Published:** 2022-01-21

**Authors:** Anzar Abbas, Bryan J Hansen, Vidya Koesmahargyo, Vijay Yadav, Paul J Rosenfield, Omkar Patil, Marissa F Dockendorf, Matthew Moyer, Lisa A Shipley, M Mercedez Perez-Rodriguez, Isaac R Galatzer-Levy

**Affiliations:** 1 AiCure New York, NY United States; 2 Merck & Co, Inc Kenilworth, NJ United States; 3 Icahn School of Medicine at Mount Sinai New York, NY United States; 4 Department of Psychiatry New York University School of Medicine New York, NY United States

**Keywords:** digital biomarkers, phenotyping, computer vision, facial expressivity, negative symptoms, vocal acoustics

## Abstract

**Background:**

Machine learning–based facial and vocal measurements have demonstrated relationships with schizophrenia diagnosis and severity. Demonstrating utility and validity of remote and automated assessments conducted outside of controlled experimental or clinical settings can facilitate scaling such measurement tools to aid in risk assessment and tracking of treatment response in populations that are difficult to engage.

**Objective:**

This study aimed to determine the accuracy of machine learning–based facial and vocal measurements acquired through automated assessments conducted remotely through smartphones.

**Methods:**

Measurements of facial and vocal characteristics including facial expressivity, vocal acoustics, and speech prevalence were assessed in 20 patients with schizophrenia over the course of 2 weeks in response to two classes of prompts previously utilized in experimental laboratory assessments: *evoked* prompts, where subjects are guided to produce specific facial expressions and speech; and *spontaneous* prompts, where subjects are presented stimuli in the form of emotionally evocative imagery and asked to freely respond. Facial and vocal measurements were assessed in relation to schizophrenia symptom severity using the Positive and Negative Syndrome Scale.

**Results:**

Vocal markers including speech prevalence, vocal jitter, fundamental frequency, and vocal intensity demonstrated specificity as markers of negative symptom severity, while measurement of facial expressivity demonstrated itself as a robust marker of overall schizophrenia symptom severity.

**Conclusions:**

Established facial and vocal measurements, collected remotely in schizophrenia patients via smartphones in response to automated task prompts, demonstrated accuracy as markers of schizophrenia symptom severity. Clinical implications are discussed.

## Introduction

Utilization of objective digital measurements of patient behavior is rapidly increasing in clinical research and practice. The development and validation of digital measurement tools in psychiatry come with both significant opportunities and risks. Significant opportunity arises as psychiatry is undergoing a paradigm shift toward the utilization of objective markers to assess illness and disease progression [1] and toward the widespread use of telehealth platforms for psychiatric care. This is particularly important when face-to-face medical care is not possible, such as during the COVID-19 pandemic [2-4].

Many behavioral and physiological markers are now accessible through digital technology such as wearables, mobile or web-based apps, and application programming interfaces [5]. Such advances hold promise in allowing new innovations in neuropsychiatry to truly scale in a manner where they can be used to develop and implement assessment and treatment for patients with significant psychiatric impairment [6].

Schizophrenia represents a poignant example of both the benefits and challenges of remote digital measurement. Clinical trials for schizophrenia drug development are often site-centric, requiring patients to appear physically at the site for measurement of disease severity. The need to travel to sites can restrict study populations to those that live in geographical proximity to the site, restricting access to participation and limiting patient diversity [7]. Current approaches for measurement of disease rely on clinician-administered measures that are costly and time-consuming to administer, leading to infrequent assessment. The instruments themselves are not well-aligned with current neurobiological definitions of illness [8].

Digital assessments address the practical challenges associated with in-person measurement of disease severity. Given that they can be administered remotely, they allow for assessments to occur in the patient’s natural environment with reduced need for in-person consultations at a clinic. Additionally, the short length of the assessments allows for them to be administered with far greater frequency than would be possible with in-person assessments. Hence, digital assessments could provide care teams greater visibility into patient health and behavior outside the clinic with the potential to inform patient responses to treatment, or the lack thereof, earlier than would otherwise be possible [9,10]. There is a need to determine the viability of such assessment to accurately measure symptom severity when deployed in real-world settings, where differentiating between significant variability and noise can pose a challenge [11-13].

A number of behavioral characteristics of schizophrenia, such as alogia (poverty of speech) and affective flattening (diminished emotional expression or emotional withdrawal) [14], can be quantified directly using standardized tasks and coding schemes [15-19], which can be automated through use of computer vision [20] and vocal acoustic [21] machine learning models. In addition to digital measures that are directly analogous to core schizophrenia symptoms, there are a number of other acoustic measures including vocal loudness, pitch variability, fundamental frequency, and jitter, which have demonstrated validity as markers of schizophrenia [16,22-24]. These markers have demonstrated specificity as measures of the negative symptom cluster, which is of particular interest given the lack of available treatment options for negative symptoms [22].

In this study, we examine the ability to measure schizophrenia symptom severity through facial and vocal analysis using videos recorded during a remote smartphone-based assessment composed of both evoked and spontaneous prompts. We compared these measures against standard clinical assessments of overall schizophrenia symptom severity (ie, total score on the Positive and Negative Syndrome Scale [PANSS]) as well as specific domains of positive (P total), negative (N total), and general (G total) symptoms, measured during in-person study visits [25]. We further conducted an exploratory analysis on the relationship between digital measures and individual symptoms of schizophrenia.

## Methods

### Participants

Individuals who had received a DSM-5 clinical diagnosis of schizophrenia or schizoaffective disorder and passed a telephone screening and were on a stable treatment regimen for atypical antipsychotic therapy for ≥2 months with no intent to change medication during the 2-week study were recruited as study participants. A total of 20 individuals, 15 with schizophrenia and 5 with schizoaffective disorder, were enrolled (8 male, 12 female) with an age range of 29 to 61 years (*µ*=45, *σ*=11). A subset of 11 individuals had their diagnosis confirmed through semistructured interviews. To be included in the study, participants needed to be able to speak, read, hear, and understand the language of the study team and the informed consent form; respond verbally to questions; follow instructions; and be willing and able to participate in all study activities, including the use of smartphones for data collection.

Given that the purpose of the study was to determine whether remote assessments would be able to appropriately collect behavioral data for assessment of disease severity in patients with schizophrenia by using digital biomarkers, data from healthy controls were not included. Data on healthy controls would have allowed for assessment of whether facial and vocal digital biomarkers can distinguish healthy individuals from patients with schizophrenia. However, we felt that past work on each of the biomarkers discussed in this paper provides sufficient evidence for this claim (Table 1).

The study was conducted at the Icahn School of Medicine’s Affective and Cognitive Therapeutics Research Lab and the protocol was approved by the Biomedical Research Alliance of New York.

### Data Collection

All study participants were assessed for severity of schizophrenia symptoms using both in-person clinical assessments and remote smartphone-based assessments over the course of the 14-day observational period. All data were collected over 3 months, from July to September 2019.

#### In-Person Clinical Assessments

The PANSS was administered in person to all participants by a trained research team member on the first (day 1) and last (day 14) of the study. For all subsequent analyses, the PANSS scores for each study participant were averaged for the 2 time points. Given the study participants were clinically stable, averaging the two PANSS scores allowed for reduction in any noise in the measurement. Multimedia Appendix 1 shows the reliability of the PANSS scores for the two time points.

#### Remote Smartphone-Based Assessments

**Figure 1:**
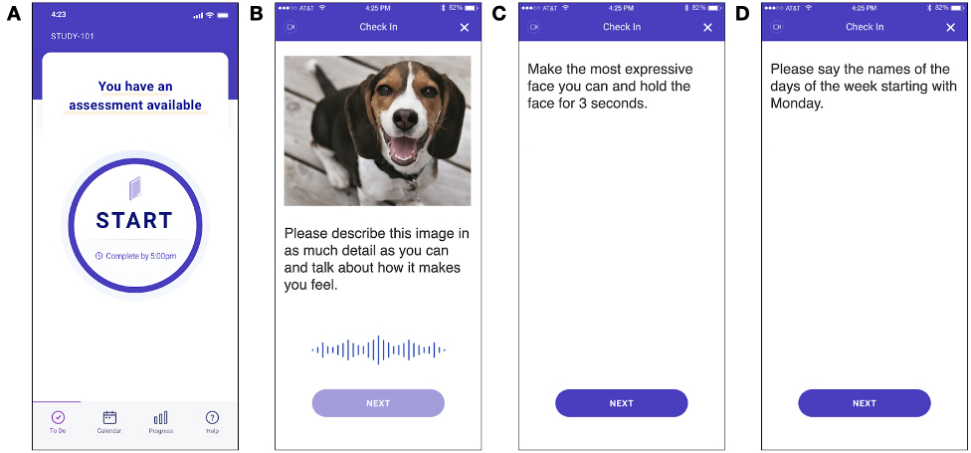
Example screenshots from the smartphone assessment all study participants took for remote and automated collection of video and audio data. During each of the prompts, the app speaks the text displayed on the screen and awaits a verbal and visual response from the participant, all while recording video and audio from the front-facing camera and microphone. (A) Screen displayed before the participant begins the assessment. (B) Prompt for collection of free behavior in response to images, showing one example image. (C) Prompt for collection of evoked facial expression behavior. (D) Prompt for collection of evoked vocal expression behavior.

On the first day of the study, all study participants were trained by a research coordinator on how to use the smartphone app [26] for remote data collection, which would capture video and audio data of participant behavior using the front-facing smartphone camera as they responded to on-screen prompts (Figure 1). This software has been used in clinical research for reporting medication adherence, electronic patient-reported outcomes, and ecological momentary assessments [27,28]. Participants were allowed to use their own smartphones or those provisioned to them by the study team for the duration of the study. The assessments were taken at scheduled time points over the course of the 14 days, and the app would send a reminder to the participant at the participant’s chosen daily reminder time when an assessment had become available. All participants received US $1 per assessment they completed using a debit card that was provided to them during study enrollment. Subjects were also compensated with US $25 for the screening visit, US $75 for the initial training, and US $200 at the final visit for device return (with an optional additional US $20 reimbursement if they used their own device, to cover data costs). The assessments were designed to capture 2 main kinds of behaviors as described below.

#### Free Speech and Spontaneous Expressivity

Participants were shown images from the Open Affective Standard Image Set [29] and asked to describe the images and talk about how they made them feel (Figure 1B). The participants’ speech and facial expressivity in response to the prompts were captured [15,16,18,19,30-32]. This assessment was conducted on days 2, 7, and 14 of the study.

#### Evoked Facial and Vocal Expressions

Participants were asked separately to make the most expressive face they could and hold it for 3 seconds (Figure 1C) and then recite the days of the week out loud (Figure 1D). These prompts were selected on the basis of prior experimental tasks used to examine emotional activity and speech in schizophrenia [31,33]. The captured video and audio were used to measure facial expressivity and acoustic characteristics of voice during the evoked expressions. These assessments were scheduled on days 1, 7, and 14 of the study.

Given that the study participants were clinically stable and maintained on the psychiatric medications they entered the study on, measurements acquired from each time point of the assessments were averaged before comparison with PANSS scores. Since we did not expect to observe significant clinical change, taking the average allows for reduction of noise and accounts for within-subject variability. Multimedia Appendix 1 shows the test-retest reliability of each of the digital measures between the 2 weeks was considerable, supporting the decision to average the measures.

### Measurement of Digital Markers

Video and audio data of participant behavior collected during the remote assessments containing protected health information (PHI) were uploaded and stored using Health Insurance Portability and Accountability Act (HIPAA)–compliant backend services. These data were then processed to extract frame-by-frame measurements of behavior, generating the first level of non-PHI data. A combination of computer vision and digital signal processing tools were used for quantification of facial and vocal behavior and subsequent derivation of visual and auditory markers of schizophrenia as described below.

All analyses were conducted using Python, along with open-source tools. All digital biomarker variables analyzed were acquired through the use of OpenDBM, an open-source software package that combines tools for measurement of facial, vocal, and movement behavior, developed partially for our study [34] and made available freely for use by all researchers.

#### Measurement of Facial Expressivity

The software library OpenFace [35] was used to measure framewise facial expressivity through quantification of action units (AUs; Multimedia Appendix 2) using a computer vision–based implementation of the Facial Action Coding System. All framewise AU measurements were normalized through division by a timepoint-specific baseline value acquired at the beginning of each assessment when the participant is not presented with any stimulus. The normalization allows for correction of any inter- and intraindividual variability; this methodology has previously been demonstrated to be necessary for measurement of facial behavior using computer vision tools and for subsequent analyses of facial expressivity [36-38]. This normalization is also necessary to account for tardive dyskinesia or other movement disorders that may be present in patients receiving antipsychotics. The time point-specific baseline normalization addresses noise in facial expressivity measurements stemming from motor abnormalities. *Facial expressivity* was calculated by taking the mean framewise intensity of all AUs over the course of the video. The method for quantifying *facial expressivity* was the same for both spontaneous and evoked expressivity. For each frame of video, OpenFace provides a confidence score denoting the likelihood that it is accurately detecting a face; only frames with a confidence score of 80% or higher were used for all downstream analyses. While OpenFace provides large amounts of information on specific AUs and emotions, in the current investigation, we focused only on *facial expressivity* because of significant evidence that patients with schizophrenia display a decrease in overall affect (eg, blunted affect) [39,40].

#### Measurement of Vocal Acoustics

The software library Parselmouth [41], which is a Python implementation of the Praat software library [42], was used for measurement of all vocal acoustic characteristics. All audio analyzed was first passed through the LogMMSE noise reduction algorithm for speech enhancement [21,43].

Despite the exploratory nature of this study and given the small data sample, we attempted to be parsimonious in the selection of markers to reduce the likelihood of false discovery. Analysis of vocal markers included those that have previously demonstrated effects in studies of individuals with schizophrenia [16,23]. Each vocal marker—calculated separately during free speech and evoked vocal expressions—include *vocal intensity*, *fundamental frequency mean*, *fundamental frequency stdev*, *vocal jitter*, *harmonics to noise ratio* and *speech prevalence* [22,24,43-45]. Descriptions of these verbal acoustic features are provided in Table 1.

**Table 1:** List of vocal acoustic variables extracted from audio files collected during participation in remote smartphone assessments and references to earlier work on their relevance in schizophrenia.

Variable	Description
*Vocal intensity*	Volume of participant’s speech, measured in decibels, which was previously shown to be decreased in individuals with schizophrenia compared to healthy controls [30].
*Fundamental frequency mean*	Average fundamental frequency of participant speech in hertz, which has been shown to be higher in individuals with schizophrenia and decreases in response to treatment [24,44].
*Fundamental frequency stdev*	SD in fundamental frequency in hertz, which has been shown to be greater in individuals with schizophrenia [24].
*Vocal jitter*	Degree of irregularity in the frequency of the participant’s speech, measured in hertz, demonstrated to be higher in individuals with schizophrenia [45].
*Speech prevalence*	Percentage of the audio file where participant speech was detected as opposed to silence; individuals with schizophrenia demonstrate increased pauses and variability in pause duration [39,46].
*Harmonics to noise ratio*	Quantification of additive noise in the participant’s speech, which has been used to predict risk of psychosis, and has shown to be correlated with symptom severity in other neurological disorders such as Parkinson disease [12,47].

### Data Analysis

Both facial expressivity and vocal characteristics were assessed during free behavior following spontaneous prompts (Table 2). Facial expressivity was also assessed during evoked facial expressions and vocal characteristics were assessed during evoked vocal expression following evoked prompts. Evaluation of vocal characteristics during the evoked expression task allowed for measurement of specific characteristics that have been previously shown to be effective measures of schizophrenia during speech (eg, *fundamental frequency mean and stdev, jitter, harmonics to noise ratio*) while also measuring speech characteristics such as amount of time spoken (ie, *speech prevalence*) [22,24,43-45]. A large number of variables can be calculated from video and audio data sources; however, the analyses presented herein were limited to features that have evidence and a theoretical basis for a relationship with schizophrenia symptom severity in the scientific literature.

**Table 2:** All variables described in Measurement of Digital Markers were calculated separately for distinct behaviors captured during the remote smartphone assessments. Each of the behaviors that were elicited and captured during the smartphone assessment and the digital markers calculated from those behaviors are listed here.

Behavior	On-screen prompt	Digital markers measured
Free behavior	*Please describe what you see in this image and talk about how it makes you feel* (Figure 1B)	Facial expressivity Fundamental frequency mean Fundamental frequency stdev Vocal jitter Harmonics to noise ratio Speech prevalence
Evoked facial expression	*Please make the most expressive face you can and hold it for 3 second*s (Figure 1C)	Facial expressivity
Evoked vocal expression	*Please say the names of the days of the week starting with Monday* (Figure 1D)	Fundamental frequency mean Fundamental frequency stdev Vocal jitter Harmonics to noise ratio Speech prevalence

#### Correlation With PANSS Subscale Scores

As the primary analysis, digital measures were correlated with overall schizophrenia symptom severity considering the PANSS total score (*PANSS Total*) along with the 3 subscales reflecting *N Total*, *P Total*, and *G Total* using Pearson’s correlation. When comparing negative symptoms, we utilized the PANSS Marder Symptom Factor, which includes two symptoms that are traditionally included in the general severity score: *Motor Retardation* and *Social Avoidance and Isolation* [48].

#### Correlation With Individual PANSS Items

As an additional exploratory analysis, digital measurements that demonstrated significance in relation to specific subscales were then further explored in relation to the specific symptoms that derive those subscales, correcting for multiple comparisons using a Benjamini-Hochberg adjusted *P* value [49]. This was an exploratory analysis conducted to further disaggregate the heterogeneity within the symptom scales to understand more specifically which clinical features were reflected in the digital measurement. The results from these analyses are provided in the supplementary materials and are not included in the main text.

## Results

Participation in the in-app remote assessments across participants was high (Multimedia Appendix 3).

### Correlation With PANSS Scores

#### Vocal Markers During Evoked Vocal Expression

Our results demonstrate that multiple digital measures are significantly correlated with overall N Total after correcting for multiple comparisons. This includes *fundamental frequency mean* (*r*=–0.64; *adjusted P*=.02), *vocal jitter* (*r*=0.56; *adjusted P*=.02), and *harmonics to noise ratio* (*r*=–0.61; *adjusted P*=.02). Two other features trended in the hypothesized direction with *P* values of <0.1 after correction for false discovery, including *speech prevalence* (*r*=–0.47; *adjusted P*=.06) and *fundamental frequency stdev* (*r*=–0.44; *adjusted P*=.07; see Table 3 for full results). Importantly, the directionality of results was consistent with prior research. For example, increased negative symptom severity was reflected in decreased speech prevalence, decreased tonal qualities of speech, and increased noise to speech sounds, consistent with the literature [16,22-24].

**Table 3:** Correlation between vocal markers during evoked vocal expression and Positive and Negative Syndrome Scale (PANSS) score showed a relationship between vocal characteristics and schizophrenia symptom severity.

Variable		Negative symptom severity	Positive symptom severity	General severity	Total	Vocal intensity	Fundamental frequency stdev	Fundamental frequency mean	Vocal jitter	Speech prevalence
**Negative symptom severity**										
	Pearson *r*	—								
	*P* value	—								
**Positive symptom severity**										
	Pearson *r*	0.452^a^	—							
	*P* value	.045	—							
**General severity**										
	Pearson *r*	0.572^b^	0.806^c^	—						
	*P* value	.008	<.001	—						
**Total**										
	Pearson *r*	0.757^c^	0.870^c^	0.947^c^	—					
	*P* value	<.001	<.001	<.001	—					
**Vocal intensity**										
	Pearson *r*	–0.091	–0.250	–0.088	–0.152	—				
	*P* value	.71	.90	.72	.64	—				
**Fundamental frequency stdev**										
	Pearson *r*	–0.436	–0.068	0.098	–0.090	–0.081	—			
	*P* value	.07	.78	.83	.71	.74	—			
**Fundamental frequency mean**										
	Pearson *r*	–0.644^a^	–0.253	–0.218	–0.373	0.475	0.577^a^	—		
	*P* value	.02	.30	.37	.70	0.10	.02	—		
**Vocal jitter**										
	Pearson *r*	0.563^a^	0.229	0.122	0.293	–0.176	–0.695^c^	–0.823^c^	—	
	*P* value	.02	.52	.93	.34	.79	<.001	<.001	—	
**Speech prevalence**										
	Pearson *r*	–0.470	–0.247	–0.292	–0.362	0.611^a^	0.043	0.781^c^	–0.373	—
	*P* value	.06	.61	.23	.38	.03	.86	<.001	.12	—
**Harmonics to noise ratio**										
	Pearson *r*	–0.610^a^	–0.195	–0.126	–0.297	0.154	0.773^c^	0.868^c^	–0.965^c^	0.422
	*P* value	.02	.51	.61	.43	.66	<.001	<.001	<.001	.07

^a^*P*<.05.

^b^*P*<.01.

^c^*P*<.001.

#### Evoked Facial Expression

*Facial expressivity* demonstrated significant relationships with the overall schizophrenia symptom severity PANSS total score (*r*=–0.71; *adjusted P*=.002) and on all PANSS subscales (N Total, *r*=–0.50; *adjusted P*=.04; P Total, *r*=–0.63; *adjusted P*=.006; G Total, *r*=–0.70; *adjusted P*=.009), in a direction consistent with the literature [15,18,19,37,38] (Table 4).

**Table 4:** Correlation between facial expressivity during evoked facial expression and the Positive and Negative Syndrome Scale score showed a relationship between facial affect and schizophrenia symptom severity.

Variable			Facial expressivity		Negative symptom severity		Positive symptom severity		General severity
**Facial expressivity**									
	Pearson *r*	—							
	*P* value	—							
**Negative symptom severity**									
	Pearson *r*	–0.500^a^		—					
	*P* value	.04		—					
**Positive symptom severity**									
	Pearson *r*	–0.628^b^		0.452^a^		—			
	*P* value	.01		.045		—			
**General severity**									
	Pearson *r*	–0.695^b^		0.572^b^		0.806^c^		—	
	*P* value	.009		0.008		<.001		—	
**Total**									
	Pearson *r*	–0.714^b^		0.757^c^		0.870^c^		0.947^c^	
	*P* value	.002		<.001		<.001		<.001	

^a^*P*<.05.

^b^*P*<.01.

^c^*P*<.001.

#### Free Behavior in Response to Images

Spontaneous measurement of vocal and facial expressions, as elicited by emotionally valenced images, demonstrated relationships between multiple vocal markers and the negative symptom cluster. Highly consistent with results of vocal measurements in response to evoked prompts, the following measures demonstrated significant relationships with N Total: *fundamental frequency mean* (*r*=–0.61; *adjusted P*=.04), *harmonics to noise ratio* (*r*=–0.58; *adjusted P*=.03), *speech prevalence* (*r*=–0.57; *adjusted P*=.03). *Vocal jitter* showed a trend in the hypothesized direction a with *P* value of <.10 (*r*=0.43; *adjusted P*=.09), and *fundamental frequency stdev* did not approach significance (Table 5). In contrast to measurement after the evoked task, *vocal intensity* measured during free behavior demonstrated significance (*r*=0.50; *adjusted P*=.05).

**Table 5:** Correlation between facial and vocal markers during free behavior and PANSS score showed a relationship between facial affect and vocal characteristics with schizophrenia symptom severity.

Variable			Negative symptom severity		Positive symptom severity		General severity		Total		Facial expressivity		Vocal intensity		Fundamental frequency mean		Fundamental frequency Stdev		Harmonics to noise ratio		Vocal jitter
**Negative symptom severity**																					
	Pearson *r*	—																			
	*P* value	—																			
**Positive symptom severity**																					
	Pearson *r*	0.452^a^		—																	
	*P* value	.045		—																	
**General severity**																					
	Pearson *r*	0.572^b^		0.806^c^		—															
	*P* value	.008		<.001		—															
**Total**																					
	Pearson *r*	0.757^c^		0.870^c^		0.947^c^		—													
	*P* value	<.001		<.001		<.001		—													
**Facial expressivity**																					
	Pearson *r*	0.142		–0.113		0.090		0.056		—											
	*P* value	.56		.64		.83		.82		—											
**Vocal intensity**																					
	Pearson *r*	–0.502^a^		–0.332		–0.225		–0.386		0.364		—									
	*P* value	.05		.17		.83		.24		.13		—									
**Fundamental frequency mean**																					
	Pearson *r*	–0.606^a^		–0.288		–0.268		–0.428		0.184		0.935^c^		—							
	*P* value	.04		.81		.27		.48		.45		<.001		—							
**Fundamental frequency stdev**																					
	Pearson *r*	–0.304		–0.189		–0.127		–0.225		0.179		0.581^b^		0.529^a^		—					
	*P* value	.24		.61		.61		.50		.46		.009		.02		—					
**Harmonics to noise ratio**																					
	Pearson *r*	–0.584^a^		–0.224		–0.097		–0.312		0.174		0.654^b^		0.774^c^		0.476^a^		—			
	*P* value	.03		.62		.97		.34		.48		.002		<.001		.04		—			
**Vocal jitter**																					
	Pearson *r*	0.426		0.147		0.015		0.194		–0.097		–0.541^a^		–0.691^b^		–0.278		–0.937^c^		—	
	*P* value	.10		.64		.95		.50		.69		.02		.001		.25		<.001		—	
**Speech prevalence**																					
	Pearson *r*	–0.567^a^		–0.260		–0.261		–0.403		0.161		0.869^c^		0.923^c^		0.260		0.575^b^		–0.510^a^	
	*P* value	.03		.66		.98		.30		.51		<.001		<.001		.28		.01		.03	

^a^*P*<.05.

^b^*P*<.01.

^c^*P*<.001.

## Discussion

### Principal Findings

In this study, we tested the hypothesis that facial and vocal markers of schizophrenia can be captured remotely in patients using brief automated smartphone-based assessments, and that such measures would be correlated to standard clinical measures of schizophrenia symptom severity. The measures show promise as objective and automated methods of assessing illness severity in the context of treatment development and decision-making. Prompts and vocal or facial measures that have previously demonstrated accuracy in controlled research settings were simplified and deployed as a brief assessment via a smartphone app in an observational study involving patients with schizophrenia. Our results support the ability to measure meaningful clinical markers of schizophrenia symptom severity via a brief smartphone-based assessment that captures data remotely and processes it through a back-end of machine learning algorithms to identify vocal and facial markers.

Our results demonstrate that vocal characteristics such as fundamental frequency, loudness, nonverbal vocal tones, and the prevalence of speech serve as specific markers of symptom severity—particularly for negative symptoms—in a direction consistent with previous literature, which used laboratory-based measures. The majority of these markers demonstrate a robust signal of negative symptom severity regardless of whether prompts were evoked or spontaneous.

The observation that vocal markers provide specificity as a metric of negative symptom severity has significant practical implications in clinical research and decision-making. Recent advances in the mechanistic understanding of negative symptoms have led to a number of promising pharmacological and cognitive treatments for negative symptoms of schizophrenia [50-53]. Such initiatives are important given the lack of US Food and Drug Administration–approved treatments for negative symptoms [54]. However, measures of negative symptoms to assess the efficacy of these treatments on the basis of objective measurement of behavior rather than subjective clinician observation are sparse [55-58].

Facial expressivity only demonstrated a relationship with schizophrenia symptom severity when captured using evoked prompts. This may indicate that either greater structure is needed to assess this marker remotely or that the prompts that were utilized were not a strong enough elicitation. Indeed, prior work has demonstrated that video rather than still images are stronger evocations to assess emotional variability in schizophrenia [59]. These findings suggest that care must be taken to determine the form of behavior from which facial expressivity is being quantified: facial expressivity during evoked prompts differs from facial expressivity during free behavior or in response to specific stimuli. Indeed, previous work has demonstrated how the context of behavior affects the measurements acquired [7]. In this study, we observed that facial expressivity in response to evoked prompts provides a robust signal for overall symptom severity.

### Limitations

This study presents a number of important limitations. While the primary hypotheses were supported, not all effects were consistent across prompts. Given the small sample size, it is impossible to conclude definitively which markers can be utilized to robustly assess schizophrenia symptom severity or impairment. Indeed, a number of relatively large correlation coefficients trended in the hypothesized direction but with *P* values of <.10, likely owing to sample size constraints. Further, despite the markers being hypothesized a priori, this work is exploratory in nature given the small sample size, limited number of assessments, and the short duration of the study. A larger assessment will be needed to replicate our findings and to assess reliability of the metrics more broadly. Additionally, the PANSS has well-documented shortcomings as a measurement tool for negative symptoms, and future work should conduct correlations with additional scales such as the Clinical Assessment Interview for Negative Symptoms or the Brief Negative Symptom Scale [60-63]. More specifically, future studies are required to individually compare specific aspects of negative symptoms with their correlates in digital measures (eg, comparison of clinician-observed blunted affect with digitally assessed facial expressivity, considering the hypothesis that greater blunted affect is correlated with reduced facial expressivity). Such studies would allow for a more direct assessment of digital assessment tools to quantify individual schizophrenia symptoms. Despite the aforementioned limitations, this study provides evidence that facial and vocal digital measures can be remotely captured in patients with schizophrenia, and that such measures demonstrate significant relationships with established measures of schizophrenia symptom severity, offering promise that these tools could be used to remotely measure and track disease severity in an objective manner.

While app-based video and audio capture utilizes a proprietary platform, this investigation utilized open-source Python-based software, available to all researchers [34]. This allows for the expansion of our study to a wider patient population, as mentioned above, and the independent validation of the methods and their implementation in this investigation by other researchers in academic and clinical research, following an open science framework for the development of digital tools for objective, accurate, and scalable measurement of disease symptoms for both mental and physical health.

### Conclusions

This study shows that facial and vocal markers, measured using computer vision and vocal analytics from video data captured remotely via a smartphone app demonstrates validity as a marker of schizophrenia and is a promising metric for negative symptom severity. Use of such technology in clinical care and clinical research settings could allow for more frequent, remotely assessed, objective measurement of disease symptoms and treatment responses in a scalable and accessible manner, which can support the development of novel treatments and risk assessment among individuals with schizophrenia.

## Appendix

Multimedia Appendix 1 Descriptive statistics for Positive and Negative Syndrome Scale scores and digital biomarkers during free behavior.

Multimedia Appendix 2 List of facial action units (AUs) whose frame-wise intensity was quantified using computer vision; AU intensities were normalized and then combined to measure facial expressivity.

Multimedia Appendix 3 Amount of participation in the naturalistic assessments deployed through the AiCure app in the duration of the study.

## Abbreviations

AU: action unit
G Total: general severity
N Total: negative symptom severity
P Total: positive symptom severity
PANSS: Positive and Negative Syndrome Scale
PHI: protected health information
