# Supplementary material for: Facial and Vocal Markers of Schizophrenia Measured Using Remote Smartphone Assessments: Observational Study
Source: JMIR Form Res. 2022 Jan 21;6(1):e26276. doi: 10.2196/26276 (PMC8817208; doi:10.2196/26276)
Supplement: Multimedia Appendix 1 [file formative_v6i1e26276_app1.docx]

**Supplementary Table 1:** Descriptive statistics for PANSS scores and digital biomarkers during free behavior.

| Item, mean (std) | Week 1 | Week 2 | Test-Retest Reliability | p-value |
| --- | --- | --- | --- | --- |
| PANSS Total | 80.5 (17.2) | 83.6 (16.2) | 0.95 | <.0001 |
| Facial expressivity | 0.107 (0.017) | 0.106 (0.023) | 0.58 | .0089 |
| Fundamental frequency mean | 74.4 (29.6) | 75.7 (26.6) | 0.75 | .0002 |
| Fundamental frequency stdev | 95.9 (18.9) | 98.8 (18.3) | 0.53 | .0021 |
| Vocal Jitter | 0.049 (0.021) | 0.05136 (0.021) | 0.85 | <.0001 |
| Harmonics-to-noise ratio | 6.32 (3.53) | 6.03 (3.42) | 0.84 | <.0001 |
| Speech prevalence | 4.89 (4.25) | 4.72 (3.99) | 0.59 | .0075 |
