# Supplementary material for: Facial and Vocal Markers of Schizophrenia Measured Using Remote Smartphone Assessments: Observational Study
Source: JMIR Form Res. 2022 Jan 21;6(1):e26276. doi: 10.2196/26276 (PMC8817208; doi:10.2196/26276)
Supplement: Multimedia Appendix 2 [file formative_v6i1e26276_app2.docx]

**Supplementary Table 1:** List of facial action units (AUs) whose frame-wise intensity was quantified using computer vision; AU intensities were normalized and then combined to measure *facial expressivity*.

| Action Unit | Description |
| --- | --- |
| AU1 | Inner brow raiser |
| AU2 | Outer brow raiser |
| AU4 | Brow lowerer |
| AU5 | Upper lid raiser |
| AU6 | Cheek raiser |
| AU7 | Lid tightener |
| AU9 | Nose wrinkler |
| AU12 | Lip corner puller |
| AU15 | Lip corner depressor |
| AU16 | Lower lip depressor |
| AU20 | Lip stretcher |
| AU23 | Lip tightener |
| AU26 | Jaw drop |
