# Supplementary material for: Facial and Vocal Markers of Schizophrenia Measured Using Remote Smartphone Assessments: Observational Study
Source: JMIR Form Res. 2022 Jan 21;6(1):e26276. doi: 10.2196/26276 (PMC8817208; doi:10.2196/26276)
Supplement: Multimedia Appendix 3 [file formative_v6i1e26276_app3.docx]

**Supplementary Table 3:** Amount of participation in the naturalistic assessments deployed through the AiCure app in the duration of the study.

| Assessment Type | Assigned | Participation |
| --- | --- | --- |
| Free speech and spontaneous expressivity | 3 | 95% |
| Evoked facial expressions | 1 | 95% |
| Evoked vocal expressions | 3 | 100% |
| Total | 7 | 97% |
